# Supplementary material for: Facile Synthesis of Aminated Graphene Quantum Dots for Promising and Selective Detection of Cobalt and Copper Ions in Aqueous Media
Source: Molecules. 2022 Nov 14;27(22):7844. doi: 10.3390/molecules27227844 (PMC9692581; doi:10.3390/molecules27227844)
Supplement: Supplementary file 1 [file molecules-27-07844-s001.zip › molecules-1917977-supplementary.pdf]

Communication

Facile Synthesis of Aminated Graphene Quantum Dots for Promising and Selective Detection of Cobalt and Copper Ions in Aqueous Media

Weitao Li [1,2,*], Ningjia Jiang [1], Luoman Zhang [1], Yongqian Chen [1,*], Jie Gao [1], Jihang Zhang [1], Baoshuo Yang [1] and Jianxin He [1,*]

[1] Textile and Garment Industry of Research Institute, Zhongyuan University of Technology, Zhengzhou 450007, China

[2] Institute of Nanochemistry and Nanobiology, School of Environmental and Chemical Engineering, Shanghai University, Shanghai 200444, China

* Correspondence: liweitao@zut.edu.cn (W.L.); chenyq@zut.edu.cn (Y.C.); hejianxin771117@163.com (J.H.)

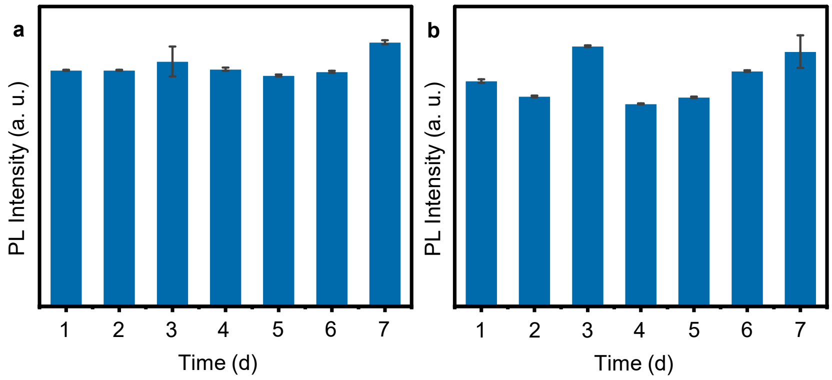

Figure S1. Fluorescence intensity comparison of GQDs (a) and N−GQDs(b) for 7 days.

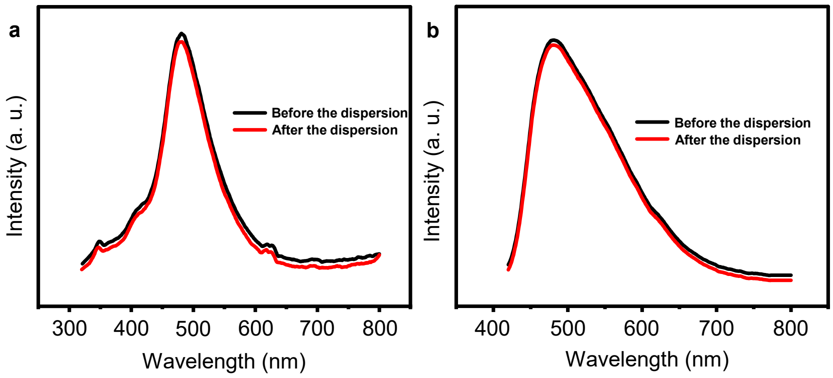

Figure S2. Fluorescence spectra of GQDs (a) and N−GQDs(b) in original solution and powder redispersion.

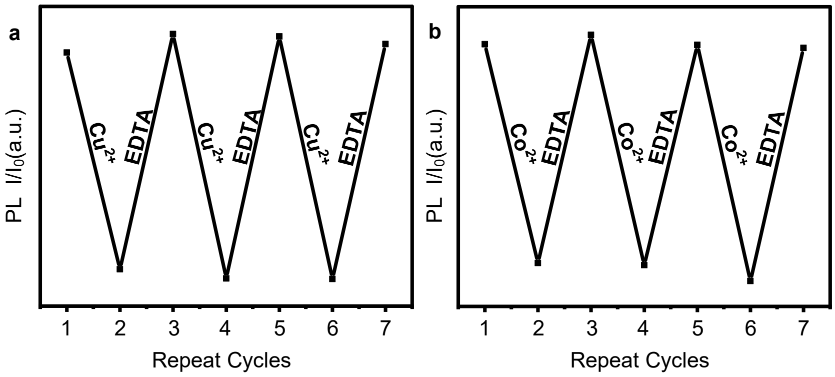

Figure S3. Fluorescence recovery cycle diagram of N−GQDS with EDTA in Cu^{2+} (a) and Co^{2+} (b) solutions.

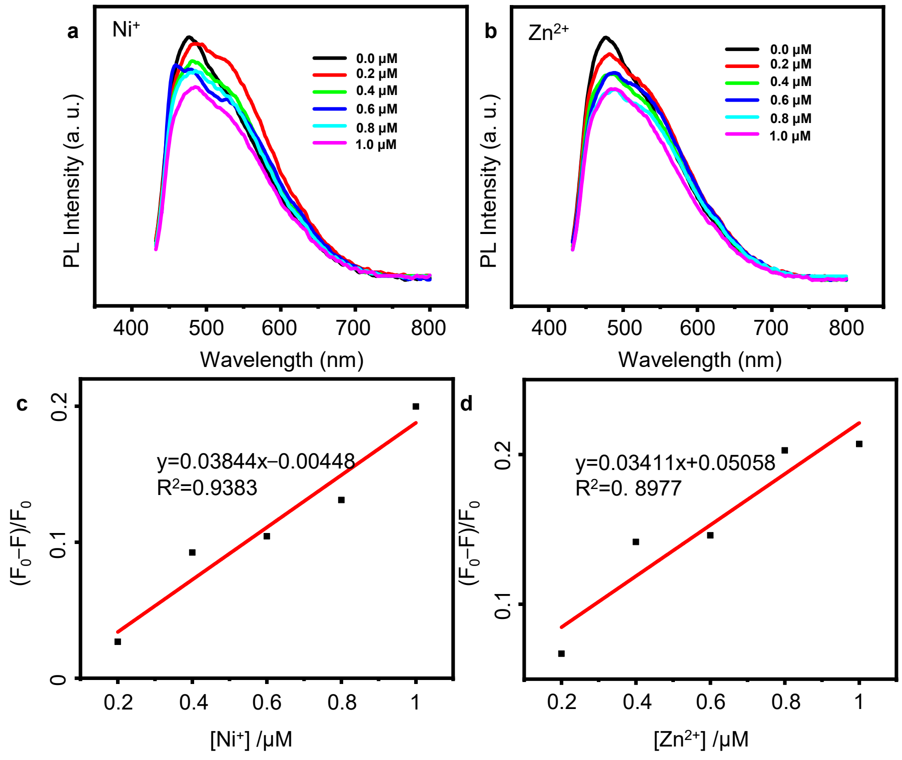

Figure S4. Fluorescence spectra of N−GQDs at different molar concentrations of Ni^{+} (a) and Zn^{2+} (b). Fitting curves of the fluorescence intensity difference ratio $(F_0−F)/F_0$ of N−GQDs for different molar concentrations of Ni^{+} (c) and Zn^{2+}(d).(Where F_0 is the fluorescence intensity of N−GQDs without adding Ni^{+}/Zn^{2+}, and F is the fluorescence intensity of N−GQDs with adding Ni^{+}/Zn^{2+})

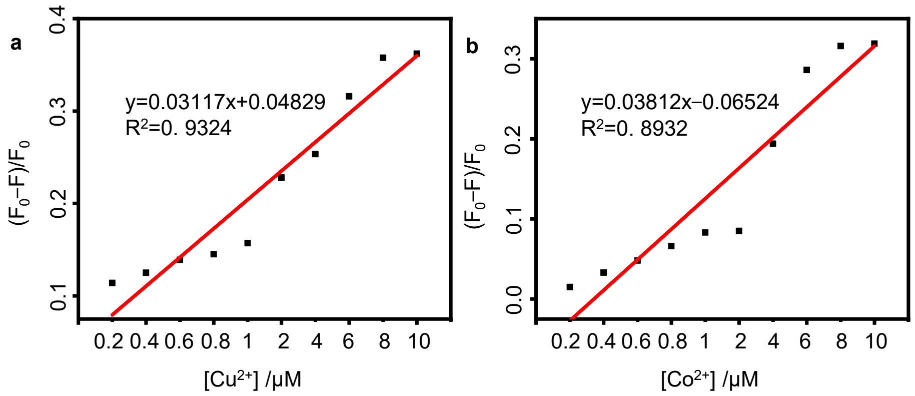

Figure S5. Fitting curves of the fluorescence intensity difference ratio $(F_0−F)/F_0$ of N−GQDs for different molar concentrations of Cu^{2+} (a) and Co^{2+}(b).(Where F_0 is the fluorescence intensity of N−GQDs without adding Cu^{2+}/ Co^{2+}, and F is the fluorescence intensity of N−GQDs with adding Cu^{2+}/ Co^{2+})
